# Functional Roles of Starch Binding Domains and Surface Binding Sites in Enzymes Involved in Starch Biosynthesis

**DOI:** 10.3389/fpls.2018.01652

**Published:** 2018-11-13

**Authors:** Casper Wilkens, Birte Svensson, Marie Sofie Møller

**Affiliations:** ^1^Enzyme Technology, Department of Bioengineering and Biomedicine, Technical University of Denmark, Lyngby, Denmark; ^2^Enzyme and Protein Chemistry, Department of Bioengineering and Biomedicine, Technical University of Denmark, Lyngby, Denmark

**Keywords:** carbohydrate binding module, surface binding site, starch synthesis, protein-carbohydrate interaction, starch binding domain, glycoside hydrolase, glycosyl transferase

## Abstract

Biosynthesis of starch is catalyzed by a cascade of enzymes. The activity of a large number of these enzymes depends on interaction with polymeric substrates via carbohydrate binding sites, which are situated outside of the catalytic site and its immediate surroundings including the substrate-binding crevice. Such secondary binding sites can belong to distinct starch binding domains (SBDs), classified as carbohydrate binding modules (CBMs), or be surface binding sites (SBSs) exposed on the surface of catalytic domains. Currently in the Carbohydrate-Active enZYmes (CAZy) database SBDs are found in 13 CBM families. Four of these families; CBM20, CBM45, CBM48, and CBM53 are represented in enzymes involved in starch biosynthesis, namely starch synthases, branching enzymes, isoamylases, glucan, water dikinases, and α-glucan phosphatases. A critical role of the SBD in activity has not been demonstrated for any of these enzymes. Among the well-characterized SBDs important for starch biosynthesis are three CBM53s of *Arabidopsis thaliana* starch synthase III, which have modest affinity. SBSs, which are overall less widespread than SBDs, have been reported in some branching enzymes, isoamylases, synthases, phosphatases, and phosphorylases active in starch biosynthesis. SBSs appear to exert roles similar to CBMs. SBSs, however, have also been shown to modulate specificity for example by discriminating the length of chains transferred by branching enzymes. Notably, the difference in rate of occurrence between SBDs and SBSs may be due to lack of awareness of SBSs. Thus, SBSs as opposed to CBMs are not recognized at the protein sequence level, which hampers their identification. Moreover, only a few SBSs in enzymes involved in starch biosynthesis have been functionally characterized, typically by structure-guided site-directed mutagenesis. The glucan phosphatase Like SEX4 2 from *A. thaliana* has two SBSs with weak affinity for β-cyclodextrin, amylose and amylopectin, which were indicated by mutational analysis to be more important than the active site for initial substrate recognition. The present review provides an update on occurrence of functional SBDs and SBSs in enzymes involved in starch biosynthesis.

## Introduction

Starch is an insoluble polymer composed of two α-glucans: the branched amylopectin, containing α-1,4-, and α-1,6-linked glucose units; and amylose, an essentially linear α-1,4-glucan. During biosynthesis starch is deposited as supramolecular, semicrystalline granules [see (Vamadevan and Bertoft, 2015) for a recent review on starch]. Despite the chemical simplicity of the two polysaccharides, starch biosynthesis is a complicated process that involves several enzyme specificities from the initial precursor formation until completion of the starch granule: ADP-glucose pyrophosphorylase, starch synthase (soluble and granule bound), starch branching enzyme, starch debranching enzyme (isoamylase), and enzymes for phosphorylation and dephosphorylation. Most of the enzymes occur in more than one isoform (see (Pfister and Zeeman, 2016; Tetlow and Emes, 2017; Goren et al., 2018) for recent reviews on the starch structure and biosynthesis) showing the same activity, but having different substrate preferences, and catalytic efficiency. In some cases, the variations may be explained by how the enzymes interact with their substrates, which might be nature’s way of fine-tuning the intricate starch granule formation processes. *In plantae* experiments have shown that a few mutations in one of the enzymes involved in starch biosynthesis can affect the structure and functional properties of the produced polysaccharides. These mutations can be situated far from the active site and may lead to loss of carbohydrate binding ability. Some enzymes possess CBMs, i.e., non-catalytic domains with carbohydrate binding sites which are connected to catalytic modules, sometimes through polypeptide linkers. The CBMs are grouped into families in the Carbohydrate-Active enZYmes (CAZy) database based on the amino acid sequence (Boraston et al., 2004; Lombard et al., 2014). Currently starch binding domains (SBDs) are found in 13 CBM families. Four of these families (CBM20, CBM45, CBM48, and CBM53) are represented in enzymes involved in starch biosynthesis, namely starch synthases, branching enzymes, isoamylases, glucan, water dikinases, and α-glucan phosphatases (Table 1). Surface binding sites (SBSs), capable of interacting with carbohydrates and exposed on the surface of catalytic domains at a certain distance from the active site or on modules intimately associated with catalytic domains (Cuyvers et al., 2011; Cockburn and Svensson, 2013; Cockburn et al., 2014) have also been identified in above mentioned enzymes (Table 1). The present review focuses on enzymes in starch biosynthesis, which have been shown experimentally to possess functional CBMs (SBDs) or SBSs.

**Table 1:** Binding data for full-length enzymes and interacting proteins involved in starch biosynthesis.

Protein	Organism	Binding site	Substrate	*K*_d_	Method	Reference
GBSSI	Barley	–	3-phosphomaltose^∗^	–	Glucan microarray	Cuesta-Seijo et al., 2016
FLO6	*O. sativa*	CBM48	Starch^∗^	–	Pull down assay	Peng et al., 2014
			Amylopectin^∗^	–		
			Amylose^∗^	–		
SSI	*H. vulgare*	SBS	Maltoheptaose	1.99 mM	SPR	Wilkens et al., 2014
			β-cyclodextrin	0.94 mM		
SSIIa	*Z. mays* L.	–	Amylopectin	3.1 mg ml^-1^	AGE	Liu et al., 2012
SSIII	*P. vulgaris* L.	3 CBM53s	Amylopectin	0.53 mg ml^-1^	AGE	Senoura et al., 2007
			Amylose	0.30 mg ml^-1^		
			Glycogen	4.04 mg ml^-1^		
			Pullulan	6.86 mg ml^-1^		
SSIV	*H. vulgare*	SBS	^∗∗^	–	–	Cuesta-Seijo et al., 2016
PTST2	*A. thaliana*	CBM48	β -cyclodextrin	1–3.3 μM	ITC	Seung et al., 2017
*St*BE1	*S. tuberosum*	–	Maltose	>50 mM	Fluorescence quenching	Mouille, 1996
			Maltotriose	11.7 mM		
			Glucosyl maltotriose	5.8 mM		
			Maltotetraose	1.1 mM		
			Maltopentaose	0.75 mM		
			Maltohexaose	0.25 mM		
			Maltoheptaose	0.16 mM		
			DP10 malto-oligo.	0.14 mM		
			DP13 malto-oligo.	0.13 mM		
			DP21 malto-oligo.	0.18 mM		
			α-cyclodextrin	6.0 mM		
			β-cyclodextrin	0.25 mM		
			γ-cyclodextrin	0.67 μM		
			Amylopectin	0.066 mg ml^-1^	AGE	
			Amylose	0.018 mg ml^-1^		
			Glycogen	>20 mg ml^-1^		
SEX4	*A. thaliana*	CBM48	Amylopectin	0.03 mg ml^-1^	AGE	Wilkens et al., 2016
			Amylose	5.42 mg ml^-1^		
			β-cyclodextrin	1.69 mM	SPR	
LSF2	*A. thaliana*	2 SBSs	Amylopectin	1.59 mg ml^-1^	AGE	Wilkens et al., 2016
			Amylose	0.68 mg ml^-1^		
			β-cyclodextrin	0.72 mM	SPR	

**Protein**	**Organism**	**Binding site**	**Substrate**	***K*_ad∗∗∗_**	**Method**	**Reference**

SSIII	*A. thaliana*	3 CBM53s	Starch	22.0 ml g^-1^	Adsorption assay	Valdez et al., 2008
SSIIIa	*O. tauri*	2 CBM53s	Starch	ND	Adsorption assay	Barchiesi et al., 2015
			Amylopectin	9.95 ml g^-1^		
			Amylose	ND		
SSIIIb	*O. tauri*	3 CBM53s	Starch	2.22 ml g^-1^	Adsorption assay	Barchiesi et al., 2015
			Amylopectin	3.84 ml g^-1^		
			Amylose	7.02 ml g^-1^		

*No binding constants were given.^∗∗^No binding detected on a glucan microarray.^∗∗∗^Adsorption constant. ND, not detected; SPR, surface plasmon resonance; AGE, affinity gel electrophoresis; ITC, isothermal titration calorimetry.

## Starch Synthases

Starch synthases (SSs; EC 2.4.1.21) catalyze transfer of glucose from the soluble precursor ADP-glucose to the non-reducing end of an α-1,4-glucan primer or growing chain. SSs belong to glycosyl transferase family 5 (GT5) and contain also a GT1 domain (Pfister and Zeeman, 2016). The GT1 domain is involved in interaction with other proteins (Seung et al., 2017). Five SSs exist: granule-bound SS (GBSS), solely responsible for biosynthesis of amylose (Tetlow and Emes, 2017), and four soluble SSs (SSI, SSII, SSIII, and SSIV) thought to be exclusively involved in amylopectin biosynthesis. In cereals the individual SSs have unique roles, each form predominantly synthesizing chains of different lengths. SSI specifically acts in synthesis of short chains in amylopectin (Delvallé et al., 2005), while SSII and SSIII have a major role in amylopectin synthesis producing chains of short to intermediate length (Zhang et al., 2005; Goren et al., 2018), and SSIV is involved in the initiation of the starch granule formation and control of the number of starch granules in the chloroplast (Roldán et al., 2007; Szydlowski et al., 2009; Crumpton-Taylor et al., 2013) (see Sections “Granule Bound SSs - SSIV”below). The different SSs use individual strategies for interacting with starch and related carbohydrates. Some have CBMs, while others have distinct SBSs. Yet other enzymes contain none of these structural features, but interact with specific proteins, which in turn interact with starch.

### Granule Bound SSs

#### Plant

Two isoforms of GBSSs belonging to GT5 are found in cereals and have no identified CBM or SBS. Barley GBSSI did not bind to starches or polysaccharides sampled in a glucan microarray analysis; the only detected binding being to 3-phosphomaltose (Cuesta-Seijo et al., 2016). Recently, however, it was shown that the localization of GBSSI to starch granules and normal amylose synthesis depended on interaction with another protein, the so-called PROTEIN TARGETING TO STARCH (PTST) 1 that is a non-catalytic protein containing coiled-coils and a CBM48. PTST interacts with the C-terminal GT1 domain of GBSS (Seung et al., 2015). PTST1 was first identified in Arabidopsis, but appears to exist in all plant species (Lohmeier-Vogel et al., 2008; Seung et al., 2015). Two additional plastidial PTSTs (PTST2 and PTST3) were identified in *Arabidopsis thaliana* leaves. PTST2 is an ortholog to the CBM48-containing FLOURY ENDOSPERM6 (FLO6) from rice (*Oryza sativa*) endosperm that influences grain starch content, granule morphology, and starch physico-chemical properties. The CBM48 of FLO6 was shown to bind to starch as well as amylopectin and amylose. FLO6 also interacts with the rice debranching enzyme isoamylase 1 that does not bind starch directly (Peng et al., 2014). PTST2 and PTST3 are proposed to interact with SSIV in Arabidopsis leaves (Seung et al., 2017), see Section “SSIV” below.

#### *Cyanobacterium* sp. CLg1

**FIGURE 1:**
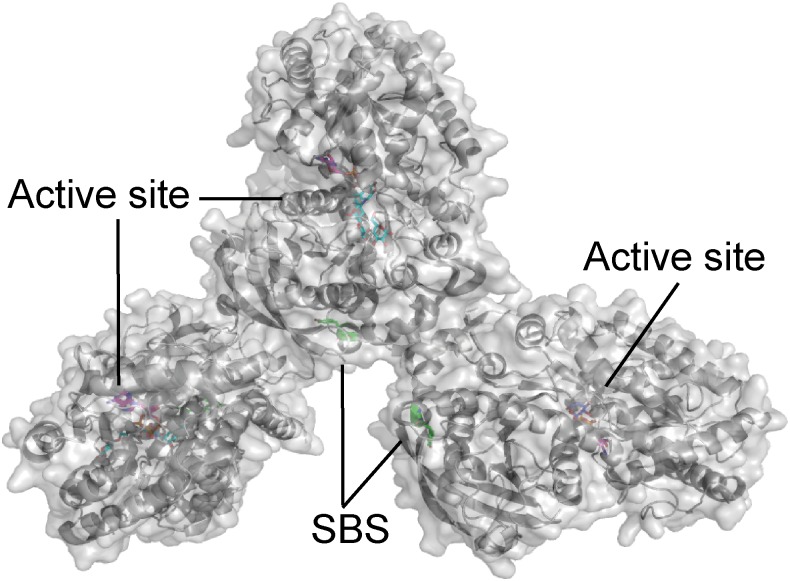
*Cyanobacterium* sp. CLg1 (CLg1GBSS) granule bound starch synthase trimer (gray) in complex with acarbose (cyan), ADP (pink) and glucose (purple), and the residues at the putative surface binding site shown as sticks (green) (PDB entry 6GNF).

Very recently, an SBS was identified in the crystal structure of the GT5 granule bound starch synthase from the *Cyanobacterium* sp. CLg1 (CLg1GBSS). CLg1GBSS crystallized as a trimer and on molecule B two planar electron densities corresponding to maltose was present out side the active site (Figure 1). At molecule A and C the putative SBS interacts with the His_6_ purification tag (Nielsen et al., 2018), which poses a steric hindrance for the maltose. However, a mutational analysis is needed to confirm the SBSs impact on CLgGBSS activity.

### Soluble SSs

#### SSI

**FIGURE 2:**
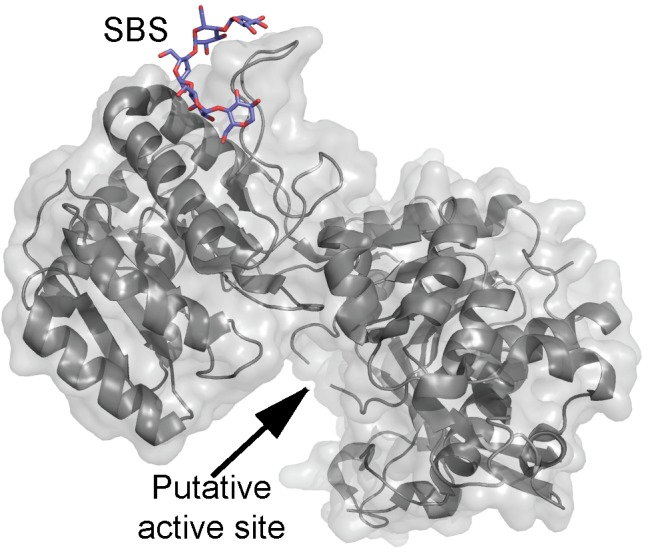
Barley soluble starch synthase I structure (gray) in complex with maltopentaose (purple) (PDB entry 4HLN).

The structure of barley SSI revealed a maltooligosaccharide-binding SBS situated 30 Å from the active site (Figure 2) and which was suggested based on mutational analysis to be involved in recognition of soluble starch and glycogen (Cuesta-Seijo et al., 2013). The SBS binds maltopentaose, -hexaose, -heptaose, and β-cyclodextrin (β-CD) as shown by surface plasmon resonance (SPR) analysis. However, *K*_d_ was only measurable for maltoheptaose and β-CD being 1.99 mM and 0.94 mM, respectively. An SBS mutant lost ability to bind β-CD and maltooligosaccharides, which indicated the SBS has higher affinity than the active site (Wilkens et al., 2014). Binding to recombinant wild type and truncated forms of SSI from maize (*Zea mays* L.) was analyzed by affinity gel electrophoresis (AGE) yielding *K*_d_ of 0.49 and 0.20 mg/ml for starch and amylopectin, respectively (Commuri and Keeling, 2001). Analysis of truncated forms and polypeptides (7–52 kDa) derived from wild type SSI by hydrolysis with trypsin indicated the N-terminal extension is neither required for catalysis nor for affinity and that a minimum size of 52 kDa (full-length is 64 kDa) was needed for catalysis and starch binding. Furthermore, removal either of approx. 50 amino acid residues from the C-terminus or a part of the N-terminal domain including a KSGGLGDV motif led to loss of activity and starch binding (Commuri and Keeling, 2001). The native SSI from maize binds amylopectin with a *K*_d_ of 2.5 mg/ml (Liu et al., 2012).

#### SSII

SSII is thought to be important for the formation of chains with intermediate lengths (DP12–25), since SSII deficiency in numerous species results in increased frequency of short glucan chains (DP6–DP11) within amylopectin, decreased abundance of DP12 to DP25 chains, and in most instances an elevated amylose to amylopectin ratio (Zhang et al., 2008). Furthermore, SSIIa in maize plays a crucial role in trafficking SSI and starch branching enzyme IIb (SBEIIb) into the granule matrix (Liu et al., 2012). The *sugary-2* mutation in maize (*Zea mays* L.), which causes major alterations to amylopectin architecture, is due to a catalytically inactive form of the endosperm-specific SSIIa having two amino acid substitutions (one outside the GT domains and one in the GT domains) and being unable to bind amylopectin. Thus, while wild type SSIIa has a *K*_d_ of 3.1 mg/ml for amylopectin no binding was observed of the *sugary-2* mutant using AGE (Liu et al., 2012).

#### SSIII

SSIIIs contain one or more CBM53s and are the best studied SSs with regard to carbohydrate binding. In the CAZy database (Lombard et al., 2014), 133 protein sequences are classified as CBM53. Most of these are found in SSIIIs, which usually have three CBM53s in tandem and therefore the number of unique proteins containing CBM53 is quite low. Three archaeal Thermococci GH enzymes each with one CBM53 are not classified in CAZy. No structure is available of CBM53 (Lombard et al., 2014). Among the SSIIIs with characterized CBM53s are SSIII from *A. thaliana* (*At*SSIII) (Valdez et al., 2008, 2011), two SSIIIs from *Osterococcus tauri* (*Ot*SSIII-A and *Ot*SSIII-B) (Barchiesi et al., 2015), and SSIII from kidney bean (*Phaseolus vulgaris* L.; *Pv*SSIII).

*At*SSIII has three N-terminal CBM53s in tandem, which preferentially bind to amylose, the first CBM53 being mainly responsible for this selective binding (Valdez et al., 2008, 2011). The second CBM53 has two binding sites, containing Y394 (binding site 1), and W366 (binding site 2), which act cooperatively with the first CBM53 in starch binding. Mutations in these sites affect kinetic parameters of *At*SSIII toward a polysaccharide substrate (Wayllace et al., 2010; Valdez et al., 2011). Recently, *in vitro* experiments showed that SBDs of *At*SSIII preferentially bind to cell wall polysaccharides over starch. Recombinantly produced *At*SSIII comprising the three *At*CBM53s thus binds to xylan and pectins with two-fold and to cellulose with 2.4-fold higher affinity than to starch (Grisolia et al., 2017). Amino acid residues at the second CBM53 were found to be important for the binding to plant cell wall polysaccharides, Y394 (binding site 1) being the most critical. Finally, transgenic plants overexpressing the three CBM53s in the cell wall are larger than wild type and have altered cell wall components (Grisolia et al., 2017).

The unicellular green alga, *O. tauri*, has three SSIII isoforms, *Ot*SSIII-A, *Ot*SSIII-B, and *Ot*SSIII-C, which according to the CAZy database contain two, three, and no CBM53, respectively (Lombard et al., 2014). Phylogenetic analysis showed that *Ot*SSIII-B is more closely related to higher plant SSIIIs, such as *At*SSIII (Barchiesi et al., 2015). The CBM53s from *Ot*SSIII-A and *Ot*SSIII-B were recombinantly produced individually and in tandem and their ability to bind starch, amylose and amylopectin was evaluated. All individual *Ot*CBM53s interacted at different levels with amylose and amylopectin in co-sedimentation assays, except for the first CBM53 from *Ot*SSIII-A that lacked both residues equivalent to those important for binding in the second *At*SSIII CBM53 (Barchiesi et al., 2015). The tandem CBM53s from *Ot*SSIII-A were able to bind amylose, but not amylopectin. Furthermore, starch binding was only observed for the first CBM53 of *Ot*SSIII-B and all three *Ot*SSIII-B CBM53s together. The results from co-sedimentation was supported by an adsorption assay using starch, amylose, or amylopectin (Barchiesi et al., 2015).

The third CBM53 from barley (*Hordeum vulgare*) SSIII (*Hv*SSIII) has been recombinantly produced and shown by glucan microarray analysis to bind to some starches, but not to any of the linear and branched oligosaccharides tested, while strong binding was observed to 3-phosphomaltose, which mimics the 3-phosphorylation of starch found *in vivo* (Cuesta-Seijo et al., 2016).

Recombinant full-length SSIII from kidney bean (*Phaseoulus vulgaris* L.) (*Pv*SSIII), as well as its N-terminal and catalytic domains on their own, have been characterized with regard to enzymatic activity and binding to amylose and amylopectin (Senoura et al., 2007). The N-terminal region of *Pv*SSIII is predicted to contain three CBM53s like other characterized SSIIIs. These N-terminal CBM53s were not essential for catalysis and had moderate effect on thermostability and pH stability. However, deletion of the CBM53s drastically decreased affinity and catalytic efficiency on glucan primers (Senoura et al., 2007). AGE indicated that N-terminal CBM53s have high affinity for amylose and amylopectin. *K*_d_ values for binding amylose, amylopectin, glycogen, and pullulan were similar for full-length *Pv*SSIII and N-terminal CBM53s alone indicating the importance of CBM53s in polysaccharide binding. *K*_d_ was in the range from 0.30 to 6.88 mg/ml for the four polysaccharides (Senoura et al., 2007).

### SSIV

**FIGURE 3:**
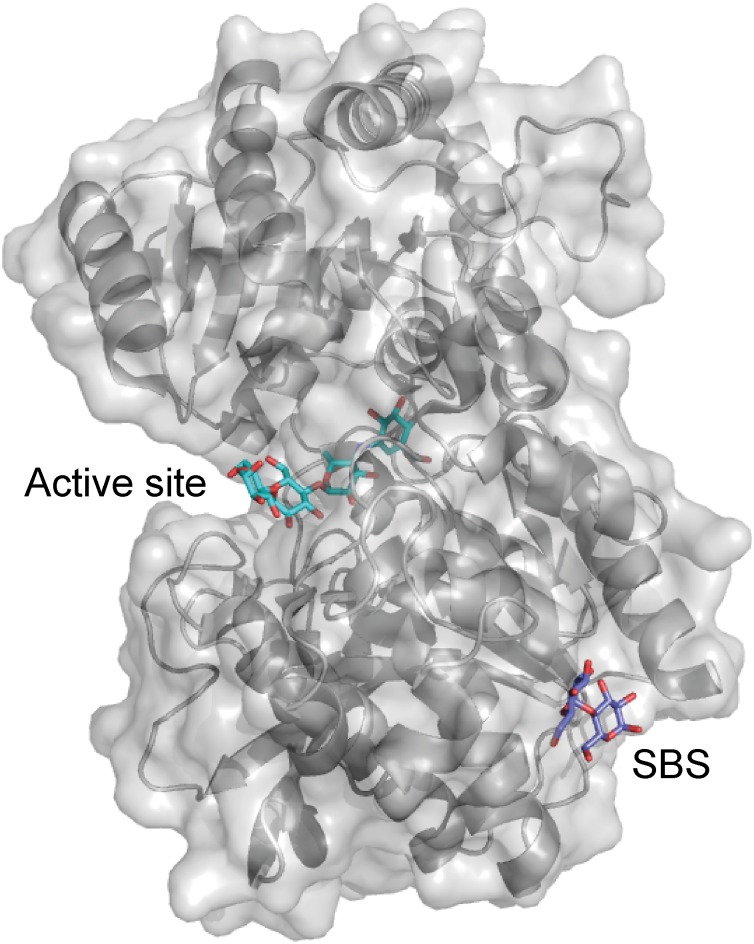
*Arabidopsis thaliana* starch synthase IV in complex with the inhibitor acarbose (cyan) and maltose (purple) (PDB entry 6GNE).

Interestingly, barley SSIV did not bind to any of the starches and polysaccharides included in a glucan microarray (Cuesta-Seijo et al., 2016). However, the non-catalytic, CBM48-containing PTST2 and PTST3 proteins are proposed to interact with SSIV in Arabidopsis leaves, and play a critical role in starch granule initiation by delivering suitable glucan primers to SSIV. PTST2 was shown by isothermal titration calorimetry (ITC) analysis to interact with β-CD (*K*_d_ ranged from 1 to 3.3 μM), and the affinity of CBM48 alone with β-CD was very similar (*K*_d_ ranged from 1.7 to 4 μM). Injection of β-CD into an equimolar mixture of PTST2 and maltoheptaose resulted in similar heat changes as when PTST2 was analyzed alone, indicating that maltoheptaose and β-CD occupy different binding sites. On the other hand, when the experiment was repeated with maltodecaose together with PTST2, significant variations were observed, while the *K*_d_ was in the same range as for PTST2 alone. This suggested that the ability of maltodecaose to adopt a structure mimicking helical amylose chains is important for binding long maltooligosaccharides to CBM48 of PTST2 (Seung et al., 2017). Very recently a crystal structure was published of GT5 *A. thaliana* starch synthase IV (*At*SSIV) in complex with the inhibitor acarbose, which showed an SBS (Figure 3) located in the same area as the SBS on the GT5 CLg1GBSS (Figure 1). AtSSIV crystallized as a dimer and the SBS was only occupied on molecule A and electron density was only present for a part of acarbose. Crystal contacts of molecule B blocked for glucan interactions (Nielsen et al., 2018). Unfortunately, a mutational analysis was not performed, so the role of the SBS remains enigimatic.

## Starch Branching Enzymes

Starch branching enzymes (SBEs; EC 2.4.1.18) catalyze formation of α-1,6-glucosidic linkages creating branch points in amylopectin and are crucial in determining structural and physical properties of starch granules (see (Tetlow and Emes, 2014) for a review on SBEs and their role in starch biosynthesis). SBEs catalyze transglycosylation reactions involving cleavage of internal α-1,4-glucosidic linkages, and transfer of products from the substrate chain toward the non-reducing end to C-6 hydroxyl groups. Multiple forms of SBEs are required for starch biosynthesis in plants and algae, unlike in glycogen synthesizing prokaryotes and eukaryotes, which have a single BE. The SBE isoforms show wide variation in chain-length transfer pattern related to their glucan substrate preferences (Tetlow and Emes, 2014). Starch BEs belong to GH13 subfamilies 8 (eukaryotic BEs; GH13_8) and 9 (bacterial BEs; GH13_9) (Stam et al., 2006); glycogen BEs are also found in GH57 (Janeček et al., 2014). GH13 BEs according to CAZy are multimodular enzymes generally including an N-terminal CBM48 (Lombard et al., 2014). Three-dimensional structures of BEs revealed the presence of both CBM48 binding sites and SBSs.

### Potato Tuber Starch BE1

A thorough analysis of *St*BE1 binding to a wide range of maltooligosaccharides by using tryptophan fluorescence quenching showed increasing affinity from maltose (*K*_d_ > 50 mM) to a maltooligosaccharide with degree of polymerization (DP) 13 (*K*_d_ of 0.13 mM) (Table 1). Notably, *K*_d_ was 0.18 mM for a maltodextrin of DP 21. It was demonstrated that maltooligosaccharides of DP below 20 were poor substrates. Binding of amylose and amylopectin yielded *K*_d_ of 0.018 and 0.066 mg ml^-1^, respectively, indicating that *St*BE1 has a substrate preference for unbranched chains. Ten-fold lower affinity for maltooligosaccharides was obtained by AGE in competition with amylose and amylopectin than by using tryptophan fluorescence quenching, which suggests the presence of a tryptophan-containing binding site different from the active site (Blennow et al., 1998).

AGE binding analysis of amylose and amylopectin to trypsin-digested *St*BE1 suggested that the C-terminal part is not important for starch binding (Blennow et al., 1998). This is surprising, because several SBSs as mentioned below are found in this region of other characterized BE1s.

### Cyanobacterial BE1

Crystal structures of a BE from *Cyanothece* sp. ATCC 51142 (*Cy*BE) in complex with maltohexaose and maltoheptaose were published recently (Hayashi et al., 2017). Unlike most cyanobacteria, *Cyanothece* sp. ATCC 51142 produces an amylopectin-like polysaccharide designated as cyanobacterial starch (Hayashi et al., 2017). Glycogen producing cyanobacteria usually have one GH13_9 BE and one GH57 BE. By contrast *Cyanothece* sp. ATCC 51142 contains three GH13_9 BEs and one GH57 BE (Suzuki et al., 2013; Suzuki and Suzuki, 2016). The three GH13_9 BE isoforms have been characterized and *Cy*BE1 and *Cy*BE2 transferred short glucans (DP 6–7), while *Cy*BE3 transfers short as well as long glucan chains (DP 30) (Suzuki et al., 2015). The chain length preferences of *Cy*BE1, *Cy*BE2 and the SBE2b isoform of rice (*O. sativa* L.) were similar, while the *Cy*BE3 specificity was similar to that of rice BE1 (Suzuki et al., 2015).

**FIGURE 4:**
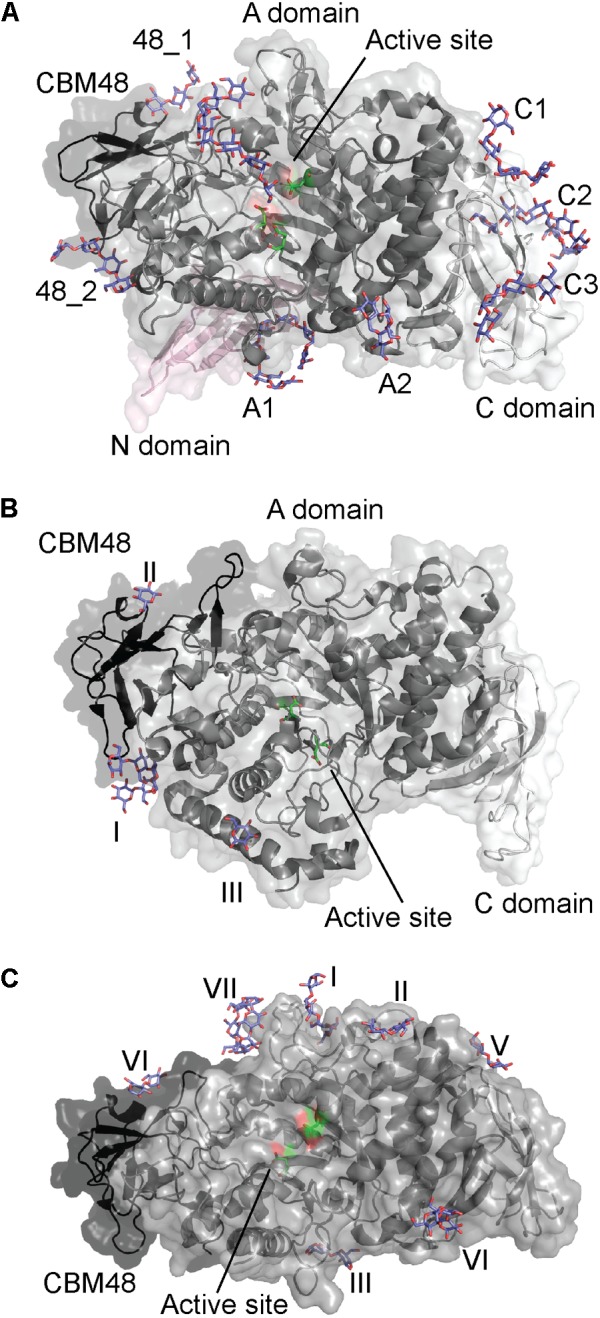
Starch branching enzymes from **(A)** *Cyanothece* sp. ATCC 51142 in complex with maltoheptaose (PDB entry 5GQX), **(B)** *O. sativa* in complex with maltopentaose (PDB entry 3VU2) and **(C)** *E. coli* in complex with maltoheptaose and α-cyclodextrin (PDB entries 4LPC and 5E6Y). The active site residues are shown as green sticks and all glucans in purple.

The crystal structure of *Cy*BE1 with maltoheptaose at the active site revealed seven additional binding sites (Figure 4A; Hayashi et al., 2017). The architecture of *Cy*BE1 was unprecedented among BEs and consists of domain N, CBM48, and the catalytic domain common for all GH13s known as domain A, and domain C (Figure 4A). Two binding sites were situated on CBM48 and the domain C contained three SBSs (Figure 4A). The functional role of these five binding sites was not investigated, but mutation of two SBSs on domain A (A1 and A2) (Figure 4A) resulted in up to 50% loss of activity with unchanged chain length profile for A1 mutants. By contrast A2 mutants showed changes in the relative proportions of the products and up to 90% decrease in activity. On the basis of these results, the maltoheptaose orientation at A1 and A2, as well as the A1 and A2 locations, the authors suggested that A1 functions as entrance for the α-glucan chain to the acceptor binding site where the new branch is transferred to the chain coming in *via* A1. A2 functions as an exit for the chain coming in *via* A1 and is also suggested to be responsible for discriminating the length of the incoming chains (Hayashi et al., 2017).

### Rice BE1

Excluding the N-domain, GH13_9 BE1 from rice (*O. sativa*; *Os*BE1) has a similar domain architecture to *Cy*BE1 (Figure 4B; Hayashi et al., 2017). The *Os*BE1 complex structure with maltopentaose reveals two binding sites on the CBM48 (Figure 4B), which are also occupied in *Cy*BE1 (Chaen et al., 2012; Hayashi et al., 2017). In addition, an SBS was observed on domain A (Figure 4B). A large number of residues interacting with maltopentaoses are conserved (Chaen et al., 2012), suggesting that this SBS has a functional role. However, mutational experiments are needed to describe the various functional roles.

### *Escherichia coli* BE1

GH13_9 BE1 from *Escherichia coli* (*Ec*BE1) resembles *Cy*BE1 in domain architecture, but does not contain the N-domain (Figure 4C; Hayashi et al., 2017). *Ec*BE1 has been crystallized in complex with α-, β-, and γ-cyclodextrins, maltohexaose and maltoheptaose, but not all ligands are seen to bind to all binding sites in *Ec*BE1. Further, *Ec*BE1 crystallized as a tetramer and the binding sites are not occupied on all four *Ec*BE1 monomers (Figure 4C). Of the seven *Ec*BE1binding sites, binding site I is at a distance of approximately 18 Å from and the closest to the active site (Feng et al., 2015, 2016).

A binding site corresponding to CBM48_1 of *Cy*BE1 was observed for *Ec*BE1 (binding site IV), while the remaining 6 sites are SBSs some of which (Figure 4C) are corresponding to A1, A2 and C1 on *Cy*BE1 (Feng et al., 2015; Hayashi et al., 2017). In addition, binding site I, occupied by linear maltooligosaccharides (Feng et al., 2015), and binding site VII, only occupied by α- and γ-CDs (Feng et al., 2016), were only seen in *Ec*BE1. For linear maltooligosaccharides to bind at binding site VII they would have to adopt a curved conformation similar to the CDs. Notably, CDs were only observed at binding sites IV–VI, which suggests that binding sites I–III are unable to accommodate the curved ligands (Feng et al., 2016). Binding sites I and II (Figure 4C) apparently accommodate maltotetraose and maltose, respectively, but their orientation suggests that they together represent a maltoheptaose molecule. Binding sites III and VI are located close to where glucan chains typically exit GH13 enzymes. Mutations in binding sites I, II, VI resulted in up to 84, 24, and 86% reduced activity, respectively (Feng et al., 2015), which clearly demonstrates their importance for the function of *Ec*BE1. Mutation of binding site VII led to loss of up to 92% activity (Feng et al., 2016).

*Ec*BE1, similarly to potato (*Solanum tuberosum*) tuber starch BE1 (*St*BE1) described above (Blennow et al., 1998), transfers maltohexaose and larger maltooligosaccharides to polymeric substrates (Binderup et al., 2000). The *Ec*BE1 active site is unoccupied in all published complex structures (Feng et al., 2015, 2016), and considered to have poor affinity for shorter linear and circular maltooligosaccharides. This probably prevents *Ec*BE1 from transferring short branches onto relatively short polymers. If this is true, the SBSs and the CBM48 binding site seem to have distinct roles. Binding sites I, II, and IV (on CBM48) could bind the glucan chain entering the active site, and III and VI could bind the glucan chain exiting the active site (Feng et al., 2015).

## Starch Debranching Enzymes

The starch debranching enzymes (DBEs) hydrolyze α-1,6-glycosidic linkages at branch points in amylopectin and oligosaccharides derived thereof. DBEs play a role during amylopectin biosynthesis and degradation. Two types of starch DBEs are found in plants, the isoamylase-type (EC 3.2.1.68) and the pullulanase-type (EC 3.2.1.41). While at least three isoforms exist of the isoamylase-type, the pullulanase-type occurs as a single form, known as limit dextrinase. Both belong to GH13 but are categorized into different subfamilies with distinct specificities; isoamylases are found in GH13 subfamily 11 (GH13_11), and pullulanase-type DBEs are found in GH13 subfamilies 12–14 (GH13_12–14) with the plant pullulanases being found solely in GH13 subfamily 13 (Møller et al., 2016). Isoamylases were hypothesized to trim misplaced branches in amylopectin, which otherwise will prevent adjacent linear chains from associating and crystallizing during the biosynthesis (Myers et al., 2000; Nakamura, 2002; Jeon et al., 2010; Goren et al., 2018). The function of pullulanase-type DBE during starch synthesis is less understood (Li et al., 2017). However, besides during germination, substantial pullulanase activity has been detected in developing rice and maize endosperms (Nakamura, 1996; Beatty et al., 1999), and the presence of mRNA for barley limit dextrinase was found in this stage of the plant lifecycle (Burton et al., 1999). A recent study has shown that variations in the gene encoding the pullulanase type DBE in *Sorghum bicolor* result in starch with better digestability (Gilding et al., 2013).

Plant DBEs are multimodular possessing at least a CBM48 in addition to the catalytic domain A and the C-domain typical of GH13. In most CBM48s from GH13_11–14 DBEs, residues predicted to constitute a canonical SBS are not fully conserved. Carbohydrate binding to CBM48 has not been demonstrated experimentally for isoamylase- and pullulanase-type DBEs (Malle et al., 2006; Mikami et al., 2006; Gourlay et al., 2009; Vester-Christensen et al., 2010; Lammerts van Bueren et al., 2011; Møller et al., 2015), but CBM48s from these enzymes possess a conserved tryptophan corresponding to Trp563 in SBS2 of a closely related CBM20 in *Aspergillus niger* glucoamylase, suggesting that plant DBE CBM48s may be intermediates between CBM20 and CBM48 (Janeček et al., 2011; Møller et al., 2016).

### Rice Isoamylase

Rice (*O. sativa*) isoamylase 1 (*Os*ISA1) does not bind starch directly, but it can interact both *in vivo* and *in vitro* with the CBM48-containing FLO6 protein (see also Section “Plant”). Hence, FLO6 might assist binding of *Os*ISA1 to starch (Peng et al., 2014). Recently, a point mutation in the barley gene *Fra* corresponding to *FLO6* was shown to cause fractured starch granules (Saito et al., 2018). These CBM48 containing proteins could be a way of the isoamylases to overcome the lack of a functional SBD.

### *Chlamydomonas reinhardtii* Isoamylase

**FIGURE 5:**
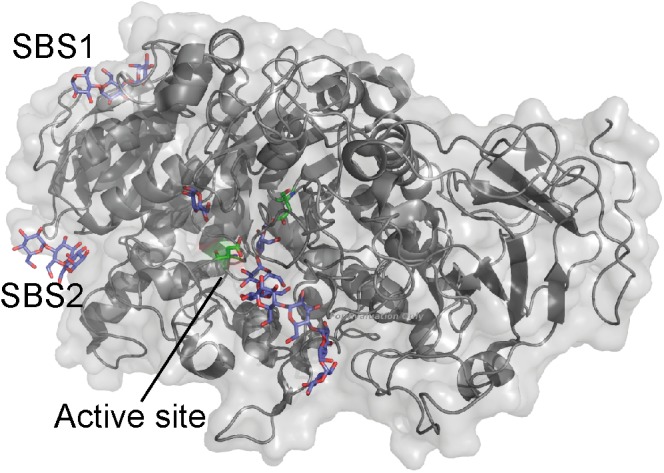
*C. reinhardtii* isoamylase A (gray) in complex with maltopentaose (purple) (PDB entry 4OKD). The active site residues are shown as sticks (green).

The green alga *Chlamydomonas reinhardtii* is a model for studying starch synthesis, and two *C. reinhardtii* isoamylases have been shown to be important for the starch synthesis (Mouille, 1996; Dauvillee et al., 2001). *C. reinhardtii* isoamylase 1 consists of the GH13 catalytic domain, a CBM48, and a characteristic GH13 C-terminal domain. The structure of the GH13 maltoheptaose-complexed *C. reinhardtii* isoamylase 1 showed carbohydrates occupying an SBS situated at the reducing-end binding area of the active site as well as a second SBS at the interface of the catalytic and C-terminal domains (Figure 5). However, the possible function of these SBSs has not been investigated (Sim et al., 2014).

## α-Glucan, Water Dikinases and α-Glucan Phosphatases

Starch phosphorylation can stimulate and hence perhaps be essential for starch biosynthesis (Hejazi et al., 2014; Skeffington et al., 2014). However, much remains to be learned about the exact metabolic role of starch phosphorylation (Mahlow et al., 2016; Xu et al., 2017). Two enzyme classes, α-glucan, water dikinases (EC 2.7.9.4) and α-glucan phosphatases (EC 3.1.3.48) are responsible for starch phosphorylation and dephosphorylation, respectively (Zeeman et al., 2010). The α-glucan, water dikinase 1 (GWD1) and GWD3 phosphorylate starch at the C6 and C3 position, respectively, and the two phosphatases, Starch Excess 4 (SEX4) and Like SEX4 2 (LSF2) dephosphorylates starch at the C6 and C3 position, respectively (Hejazi et al., 2010; Santelia et al., 2011).

### GWD1

GWD1 from *S. tuberosum* (*St*GWD1) has a C-terminal catalytic domain and two CBM45s: CBM45-1 located at the N-terminal next to the chloroplast transit peptide and CBM45-2 at the center of the sequence, and large areas of the sequence were unmapped (Glaring et al., 2011). Despite the plethora of sequence data available since 2011, a new search against the Conserved Domains Database (Marchler-Bauer et al., 2017) did not reveal additional domains in the unmapped regions.

Each of the *St*GWD1 CBM45-1 and CBM45-2 was produced recombinantly, but only CBM45-2 was stable in solution and only at pH 8. SPR and ITC showed low affinity of CBM45-2 for the starch model β-CD (SPR: *K*_d_ = 0.38 mM; ITC: *K*_d_ = 0.68 mM). Such low affinity may be a prerequisite for a dynamic interaction of GWD1 with the starch granule facilitating a necessary tight control of the starch metabolism (Glaring et al., 2011). *St*GWD1 CBM45-1 was prepared by trypsin hydrolysis of the full-length enzyme and showed relatively weak affinity, yielding *K*_d_ of 7.2 mg ml^-1^ for starch granules in an adsorption assay and *K*_d_ of 1.2 mg ml^-1^ for soluble starch by using AGE (Mikkelsen et al., 2006). Mutations in the binding site of CBM45-1 of full-length *St*GWD1 resulted in complete loss of binding for these starch substrates demonstrating its pivotal role in enzyme function (Mikkelsen et al., 2006).

### GWD3

GWD3 from *A. thaliana* (*At*GWD3) has a single CBM20 appended at the N-terminus and a C-terminal GWD catalytic domain, while the function of the ∼700 amino acid residues long segment in between these two domains is unknown (Janeček et al., 2011). As for GWD1, search against the Conserved Domains Database (Marchler-Bauer et al., 2017) failed to identify any additional domains. Most CBM20s have two binding sites (Janeček et al., 2011), but presumably only binding site 1 is functional in *At*GWD3 CBM20, which lacks residues typically for binding site 2 (Christiansen et al., 2009a).

Compared to CBM20s from amylolytic enzymes having *K*_d_ for β-CD in the μM-range recombinant *At*GWD3 CBM20 showed unusually low affinity for α-, β-, and γ-CDs (*K*_d_ of 0.22–0.84 mM) as determined by SPR (Christiansen et al., 2009a). As for CBM45-2 of *St*GWD1, this may allow dynamic binding of *At*GWD3 CBM20 facilitating GWD3 regulation (Christiansen et al., 2009b). The binding of α-, β-, and γ-CD by *At*GWD3 CBM20 depended on pH – e.g., *K*_d_ for γ-CD was 0.84 and 5.56 mM at pH 5.5 and pH 9, respectively. However, for β-CD the observed affinity increased from *K*_d_ of 1.09 mM at pH 6 to 0.53 mM at pH 9, and this pH dependence was suggested to be related to the plants physiological needs as the binding would be stronger during the day when stromal pH increases (Christiansen et al., 2009a).

### Starch Excess 4

**FIGURE 6:**
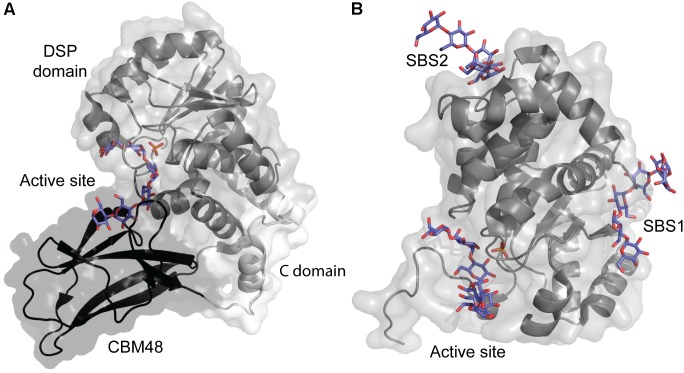
**(A)** *A. thaliana* Starch Excess 4 in complex with maltoheptaose (purple) and phosphate in the active site (orange) (PDB entry 4PYH). **(B)** *A. thaliana* Like SEX4 2 (gray) in complex with maltohexaose (purple) and phosphate in the active site (orange) (PDB entry 4KYR).

The crystal structure of *A. thaliana* SEX4 contains a single maltoheptaose molecule that spans the extended binding pocket at the interface of the catalytic dual-specificity phosphatase (DSP) and CBM48 domains (Figure 6A; Meekins et al., 2014). *K*_d_ of *A. thaliana* SEX4 for amylose and amylopectin was determined by AGE to be 5.42 and 0.03 mg ml^-1^, respectively, in agreement with the preference of SEX4 for double helical structures in amylopectin. SPR analysis gave *K*_d_ for β-CD of 1.69 mM and a stoichiometry of 2.89, indicating three β-CD binding sites on SEX4, although this remains to be confirmed by structural analysis (Wilkens et al., 2016). Mutation of residues responsible for binding in both domains resulted in 10–80% loss of activity toward *para*-nitrophenyl phosphate (Meekins et al., 2014). Furthermore, the CBM48 mutant lost 95% and a DSP mutant 76% of the amylopectin binding affinity showing that the CBM48 plays a critical role in the function of SEX4 (Wilkens et al., 2016).

### Like SEX4 2

Three distinct maltohexaose binding sites are seen in a crystal structure of *A. thaliana* Like SEX4 2 (LSF2) (Figure 6B). Of these, two SBSs, SBS1 and SBS2, are situated > 20 Å away from the active site which accommodates the third maltohexose molecule (Meekins et al., 2013). Using AGE *A. thaliana* LSF2 was shown to have *K*_d_ of 0.68 and 1.59 mg ml^-1^ for amylose and amylopectin, respectively, emphasizing its preference for the single chain helical conformation in amylose (Wilkens et al., 2016). LSF2 specifically hydrolyses C3 phosphate ester groups in starch (Santelia et al., 2011), which are hypothesized to disrupt the double helical conformation in amylopectin (Blennow and Engelsen, 2010). The higher affinity of LSF2 for amylose thus mimics its natural substrate preference (Wilkens et al., 2016). Unexpectedly, despite the long distance to the active site, SBS1 and SBS2 mutations resulted in 50 and 88% loss of activity for *para*-nitrophenyl phosphate, respectively (Meekins et al., 2013). Remarkably, however, molecular dynamics simulation showed that binding at the two SBSs affected the active site thus supporting the role of SBSs in activity. Mutation at all three binding sites reduced affinity for amylose below a measurable level. Still, comparison of the mobility of SBSs mutants in AGE pointed to their involvement in starch binding. As SBS2 seemed to have higher affinity for amylose than SBS1, it was suggested to be responsible for the initial starch recognition (Wilkens et al., 2016).

## Conclusion and Looking Ahead

As the presence of either CBMs or SBSs in several cases has been crucial for correct starch biosynthesis, the recent focus on non-catalytic sites can potentially open new promising routes to obtain useful modified starches.

Current insights on CBM53s from SSIIIs and their interaction with starch may be useful for manipulating catalytic efficiency of SSIII by altering the active site structure without modifying the catalytic module. Determination of the structure of a CBM53 would take the knowledge to the next level and improve understanding of the protein–carbohydrate interactions, maybe to deduce a mechanism for how the different CBM53s can alter activity of SSIII (Wayllace et al., 2010).

SBSs in BE1s appear to define the length of transferred branch chains (Blennow et al., 1998; Feng et al., 2015; Hayashi et al., 2017), which could potentially constitute a target for rational protein engineering. Recently, the non-catalytic proteins PTST2 and FLO6 gained attention as targets for obtaining modified starches. The *PTST* gene has been demonstrated to be essential for amylose synthesis in *A. thaliana* leaves and could be useful in obtaining amylose-free starches, which find extensive applications in food and non-food industrial products (Santelia and Zeeman, 2011; Seung et al., 2015). CBM48 of PTST2 (FLO6) was suggested as a potential target for biotechnological modification of starch, particularly for modifying granule size. A *ptst2 A. thaliana* mutant had larger starch granules, but with similar morphology to wild type granules, while much smaller granules were observed in the *ptst2* overexpression lines (Seung et al., 2017). Furthermore, a knock-out of FLO6 in rice was shown to affect starch synthesis (Peng et al., 2014).

Notably, these various examples evidently demonstrate that properties of starch can be modified by altering the enzyme–substrate interaction during starch biosynthesis. Yet, the impact of especially SBSs on the function of the starch biosynthetic enzymes is still to be further explored. However, the challenge is that they are not easily recognized based on bioinformatics analysis, like the carbohydrate binding sites of CBMs.

## Author Contributions

MM, CW, and BS conceptualized the manuscript, which was written by CW and MM with invaluable input from BS.

## Conflict of Interest Statement

The authors declare that the research was conducted in the absence of any commercial or financial relationships that could be construed as a potential conflict of interest.
